# Dynamic Characteristic Analysis of Antibodies in Patients With COVID-19: A 13-Month Study

**DOI:** 10.3389/fimmu.2021.708184

**Published:** 2021-07-20

**Authors:** Danrong Shi, Tianhao Weng, Jie Wu, Chunyan Dai, Rui Luo, Keda Chen, Miaojin Zhu, Xiangyun Lu, Linfang Cheng, Qiuqiang Chen, Fumin Liu, Zhigang Wu, Haibo Wu, Changzhong Jin, Miao Guo, Zhe Chen, Nanping Wu, Hangping Yao, Min Zheng

**Affiliations:** ^1^ State Key Laboratory for Diagnosis and Treatment of Infectious Diseases, National Clinical Research Center for Infectious Diseases, Collaborative Innovation Center for Diagnosis and Treatment of Infectious Diseases, The First Affiliated Hospital, School of Medicine, Zhejiang University, Hangzhou, China; ^2^ Key Laboratory of Digestive Pathophysiology of Zhejiang Province, The First Affiliated Hospital of Zhejiang Chinese Medicine, Zhejiang Chinese Medical University, Hangzhou, China; ^3^ Shulan International Medical College, Zhejiang Shuren University, Hangzhou, China; ^4^ Reagent R&D Department, Hangzhou Chemi Health Technology Co., Ltd., Hangzhou, China; ^5^ Information Engineering Institute, Hangzhou Dianzi University, Hangzhou, China

**Keywords:** SARS-CoV-2, immunoreaction, spike glycoprotein, receptor binding domain, nucleocapsid protein, neutralizing antibodies

## Abstract

There is a worldwide pandemic of Severe acute respiratory syndrome coronavirus 2 (SARS-CoV-2) infection; yet our understanding remains limited on the characteristic of antibodies, especially for dynamic long-term tracking. Sequential serum samples were collected up to 416 days post onset of symptoms (POS) from 102 patients who were hospitalized with coronavirus disease 2019 (COVID-19). Immunoglobulin (Ig)G, IgM, and IgA levels targeting SARS-CoV-2 spike 1 receptor-binding domain (S1-RBD), spike 2 extracellular domain (S2-ECD), and nucleocapsid protein (N) were quantified as well as neutralizing activity. We were pleasantly surprised to find that the antibody remained detective and effective for more than a year POS. We also found the varied reactions of different antibodies as time passed: N-IgA rose most rapidly in the early stage of infection, while S2-IgG was present at a high level in the long time of observation. This study described the long traceable antibody response of the COVID-19 and offered hints about targets to screen for postinfectious immunity and for vaccination development of SARS-CoV-2.

## Introduction

The novel Coronavirus, severe acute respiratory syndrome coronavirus 2 (SARS-CoV-2), has resulted in a worldwide pandemic, causing serious public health crises and economic losses. SARS-CoV-2 is the seventh type of coronavirus including severe acute respiratory syndrome coronavirus (SARS-CoV) and middle east respiratory syndrome coronavirus (MERS-CoV) to infect humans, it can lead to symptoms ranging from asymptomatic to fever, cough, myalgia, diarrhea, dyspnea, or even death (1–3).

SARS-CoV-2 is a single-stranded RNA virus encoding several vital structural proteins: spike (S) glycoprotein, nucleocapsid (N) protein, membrane (M) protein, and envelope (E) protein. Antibodies targeting N and S, including immunoglobulin (Ig)G, IgM, and IgA, can be detected generally (4, 5), and seroconversion from negative to positive in the majority of patients infected with SARS-CoV-2 occurs 7 to 14 d post onset of symptoms (POS) (6). S is located on the surface of the virion and comprises S1 and S2 subunits, and a transmembrane (TM) segment. S1 has been confirmed to recognize and bind to the angiotensin-converting enzyme 2 (ACE2) on host cells through its receptor-binding domain (RBD) (7–10) followed by fusion of viral and cell membranes mediated by S2, and then the virus genome is released into cell (11, 12). The RBD segment of S is highly immunogenic, antibodies and inhibitors targeting the RBD can block the virus contact with ACE2, thus producing an antiviral effect (10, 13, 14).

The humoral immune response is the key to clearing the cytopathic virus and is the main part of the memory response to prevent re-infection, also an important clue to control the infectious disease. Thus, there is an urgent need for a long-term serological study to evaluate the extent and duration of immunity in SARS-CoV-2. In previous studies, patients with COVID-19 had neutralizing antibodies (Nab) correlated with anti-S1, RBD, N, and S2 antibodies, which remained relatively stable for 5 months POS and were higher in older and male people who had more serious clinical condition than in younger and female people (5, 15–18). In a recent published study from Wuhan, China (19), neutralizing antibody levels maintained stable for 9 months and the positive rate of IgG did not significantly decrease although titers decreased. However, no studies have described the long-term response of specific antibodies in SARS-CoV-2 infected patients longer than 1 year. Importantly, the duration and effect of antibodies and their ability to resist reinfection are unclear.

To assist screening and research of SARS-CoV-2, abundant efforts have been made to develop highly accurate diagnostic tests, including immunological antibody detection (20). The quantum dot (QD)-labeled lateral flow immunochromatographic assay (LFIA) is a point-of-care test (POCT) that is able to detect SARS-CoV-2 antibodies or antigens within 5-30 minutes. Multiple quantum dots embedded in organic polymers can further amplify the fluorescence signal of quantum dots (the fluorescence intensity of quantum dots nanobeads at the same number of moles is 2863 times that of quantum dots), thus significantly improving the sensitivity of immunodetection. The LFIA we used is also based on the double antibody sandwich method and the signal intensity is proportional to the antibody concentration to be detected analogous to enzyme linked immunosorbent assay (ELISA) and chemiluminescent immunoassay (CLIA). A QD-labeled LFIA strip can obtain quantitative data using a portable fluorescence detector, enabling analysis of the dynamic information of antibodies.

In this present study, we employed a quick antibody detection assay based on QD-labeled LFIA to measure the dynamic level of SARS-CoV-2 specific antibodies for exceed 1 year, including IgG, IgA, and IgM targeting S1-RBD, S2-extracellular domain (ECD), and N. Then, we measured the neutralizing activity of the same sample of serum using live SARS-CoV-2 and analyzed the association of antibody level, neutralizing titers, and clinical conditions. We also established a Random Forest model to predict neutralizing activity from tested antibodies. Overall, this study gives strong evidence that antibodies in inpatient are still detectable and maintained neutralizing activity 1 year after infection with COVID-19, and provides novel insights into target selection in antibody detection and vaccination research.

## Materials and Methods

### Sample Collection

The present study included 102 individuals who were confirmed with SARS-CoV-2 infection by quantitative polymerase chain reaction (qPCR) and were inpatient between January 19, 2020 and March 12, 2020 in the first affiliated hospital of Zhejiang university, school of medicine, Hangzhou, China. Severity types were inferred from the Diagnosis and Treatment Protocol for Novel Coronavirus Pneumonia of China. Blood samples were taken from 2 to 416 d POS during the hospital stay and during long follow-up after discharge. The number of patients and serum during each follow-up period were shown in **Table S1**. Data on the patient information and clinical conditions were extracted from hospital electronic medical record system.

The Ethics Committee of The First Affiliated Hospital, Zhejiang University School of Medicine, approved the present study (ethical approval no. 2020IIT-255). Since archived specimens were used, patient consent statement was waived.

### Virus and Cells

The virus isolates (nCoV-19/Hangzhou/ZJU-01 to ZJU-11/2020) were separated from clinical samples of qPCR-confirmed SARS-CoV-2 patients in the First Affiliated Hospital of Zhejiang University School of Medicine. Vero cells (African green monkey kidney, ATCC CCL-81) were cultured in Minimum Essential Medium (MEM) (Life Technologies, Carlsbad, CA, USA) supplemented with 5% fetal bovine serum (Life Technologies) in a 5% CO_2_ incubator at 35°C. SARS-CoV-2 was passaged in Vero cells and the virus stock was titrated by plaque assay. All experiments involving infectious virus were conducted in an approved biosafety level III laboratory (CNAS BL0022, National Key Laboratory of infectious diseases diagnosis and treatment, Zhejiang University).

### Antibody Measurement Using QD-LFIA

The QD-LFIA we used selected S1-RBD (Novoprotein, Shanghai, China), S2-ECD (Sino Biological, Beijing, China), and N (Winbio, Xiamen, China), labeled with QD nanobeads (NanoGen, Beijing, China) respectively, as detection antigens to measure the specific immunoglobulins (IgG, IgM, IgA) in serum. The corresponding mouse anti-IgG (Winbio), anti-IgM (Ebiocore, Hangzhou, China), and anti-IgA (Ebiocore) and quality control antibody goat anti-rabbit IgG (Clongene Biotech, Hangzhou, China) were coated on a nitrocellulose membrane as the capture antibodies. The strip assay was manufactured by *Hangzhou Chemi Health Technology Co., Ltd.,China.* Serum samples were diluted 100 times with loading buffer (0.02M PBS+0.1%BSA+0.125%Tween20+0.15%proclin300, ph7.4) and were added into the well on the assay strips. Clinically collected serum samples which were SARS-CoV-2 negative was used as negative control, and a serum sample from a COVID-19 patient which was confirmed by enzyme-linked immunosorbent assay (ELISA) to contain all the types of antibodies was used as a positive control. After 10 minutes, the strips were read using a portable fluorescence detector (Helmen, Suzhou, China). The results were in the form of T (test) and C (control) ratio.

### *In Vitro* Microneutralization Assay

Serum samples from patients were diluted in MEM culture medium (5% fetal bonus serum) in series from 1:10 to 1:1280 with 2 times gradient in 96-well plate (Greiner Bio-One, Germany). Clinically collected serum samples which were SARS-CoV-2 negative was used as negative control, and an anti-SARS-CoV-2 RBD antibody P17 which has been proved to have significant anti-SARS-CoV-2 effect in our previous study (21) as positive control. Diluted serum samples (50 μl) were mixed with 50 μl of 200 TCID_50_ SARS-CoV-2 virus (the final serum dilutability was from 1:20 to 1:2560 with 2 times gradient), and placed at 37°C for two hours. Then, 100 μl of Vero cells were added (10^4^ cells per well), and incubated in 5% CO_2_ at 35°C. The final result was confirmed by cytopathic effect (CPE) observation on day 6 of culture. An effective test should meet the following requirements: the serum itself has no obvious cytotoxicity; negative control was established and its CPE reached ++++; positive control was effective and had no CPE. The antiviral effective concentration or titer of the serum is the lowest antibody concentration or the highest antibody dilution that can inhibit the CPE caused by SARS-CoV-2 infection with 100 TCID_50_.

Three serum samples from three patients were used initially to verify the Nab activities against 11 isolates of SARS-CoV-2 (ZJU-01 to ZJU-11). ZJU-05 was chosen to test all the serum samples.

### Immunofluorescence Microscopy

Immunofluorescence was used to further verify the neutralization test. Vero cells in plate were washed with phosphate-buffered saline (PBS), fixed in 80% precooled acetone (Sigma-Aldrich, USA) for 30 min, then washed and blocked in 1% bovine serum albumin at room temperature (19-21°C) for 30 min, and then incubated with anti-Spike RBD Rabbit monoclonal antibodies (mAbs) (1:1000; Sino Biological) at 4°C overnight. The plates were washed and added with Alexa Fluor488^®^-conjugated Goat Anti-rabbit IgG secondary antibody (1:1500; Abcam, Cambridge, UK) at room temperature for 2 hours. 4’,6-Diamidino-2-Phenylindole, Dihydrochloride (DAPI) (2 µM; Abcam) was used to stain the nuclei (**Figure S1**).

### Statistical Analysis

Temporal changes of antibodies were plotted using locally weighted scatterplot smoothing (LOWESS), and the differences in the temporal changes of antibodies could be examined using the 95% confidence interval belt. The cumulative seroconversion rate was analyzed using the Kaplan–Meier method and log rank test. In addition, a Heatmap was employed for visualization of the proportion of detectable antibodies over time since illness onset. We also plotted Box-plots to comparing antibody concentrations in the different categories. Student’s t tests were used to analyze differences in mean values between groups. A correlation matrix was used to examine the correlation coefficients between Nabs and antibodies, as well as the Nab activities within the 11 isolates of SARS-CoV-2 in three patients. All data analysis was performed using software R, version 3.6.3. A. P value < 0.05 was considered statistically significant. All data visualizations were produced using ggplot2. Finally, we constructed a random Forest plot to predict the Nab titers (divided by 1:20) from the tested immunoglobulins (function interface: https://scikit-learn.org/stable/modules/generated/sklearn.ensemble.RandomForestClassifier.html; decision tree source code: https://github.com/scikit-learn/scikit-learn/blob/42aff4e2e/sklearn/ensemble/_forest.py#L884).

## Results

### One-Year Dynamic Changes in Specific Antibody Responses to SARS-CoV-2

To understand the post infectious immunity, we tested the RBD-, S2-, N- specific IgG, IgM and IgA in sequential serum samples from 102 patients with qPCR-confirmed SARS-CoV-2 infection between 2 to 416 d POS using QD-labeled LFIA, and measured the neutralizing activity to live SARS-CoV-2 from the same sample. The demographic characteristics were showed in **Table 1** and **Figure S2**. All patients in the study were discharged or transferred after two consecutive negative qPCR tests, and there were no reported deaths fortunately. Serum samples from 100 individuals who were SARS-CoV-2 negative were used to determine the cut off value and estimate the specificity of the QD-labeled LFIA, results were showed in **Table S2**. Seroconversion was defined as a change from antibody seronegative to seropositive.

**Table 1:** Demographic characteristics of patients infected with SARS-CoV-2.

Gender, No. (%)	
Male	62 (60.8%)
Female	40 (39.2%)
**Age, median. (IQR)**	55 (44.8–65.3)
**Severity, No. (%)**	
Mild	2 (2.0%)
Moderate	25 (24.5%)
Severe	45 (44.1%)
Critically ill	30 (29.4%)
**Virus shedding time, median (IQR)**	17.5 (13–25)
**Follow-up**	
Range of day POS	2–416
Sampling times, median (IQR)	6 (4–8)

IQR, interquartile range; No, number; POS, post onset of symptoms.

**Figure 1:**
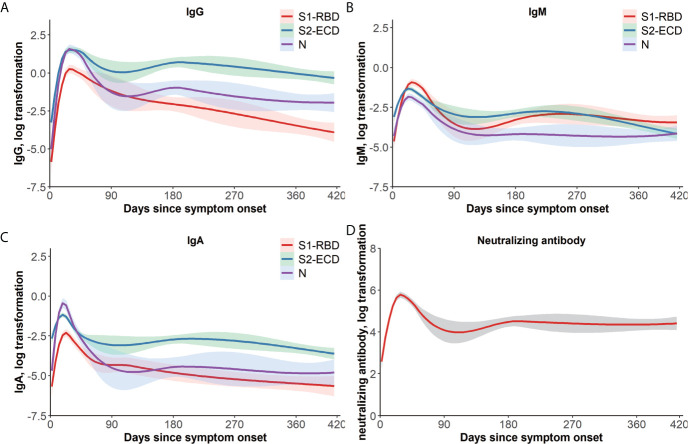
One-year dynamic changes of immunoglobulins and Nab titers. LOWESS fitting curves of S1-RBD, S2-ECD, and N-specific IgG **(A)**, IgM **(B)**, and IgA **(C)**. Days since symptom onset form the x-axis and log transformation of antibodies T/C value tested by a QD-labeled LFIA form the y-axis, and the differences of temporal change of antibodies could be examined by the 95% confidence intervals belt. Red, green, and purple curves represent S1-RBD, S2-ECD, and N, respectively. **(D)** LOWESS fitting curves of Nab titers over time. Days since symptom onset form the x-axis and log transformation of Nab titers form the y-axis. The dashed belt indicates 95% confidence intervals. ECD, extracellular domain; Ig, immunoglobulin; LFIA, lateral flow immunochromatographic assay; LOWESS, locally weighted scatterplot smoothing; N, nucleocapsid protein; Nab, neutralizing antibodies; QD, quantum dot; RBD, receptor-binding domain; S, spike; T/C, test/control.

The dynamic curves of the specific IgG, IgM, and IgA antibodies targeting S1-RBD, S2-ECD, and N responses varied (**Figures 1A–C**). In general, the IgG level remained a relatively high level in the long follow-up duration. Specifically, S2-IgG reacted most rapidly and was maintained at a high level during the whole observation period, followed by N-IgG and S1-RBD-IgG. IgM levels of T/C against all three target antigens were lower than those of IgG and IgA, and decreased rapidly. N-IgM was maintained at the lowest level during the observation period. For IgA, S2-IgA also remained a high level for the longest time. In contrast, N-IgA increased rapidly in the early days POS and reached a highest peak, then decreased markedly. The time to peak of the three antigens of three immunoglobulins and Nab (**Figure 1D**) were all about 15–30 days and were generally consistent.

**Figure 2:**
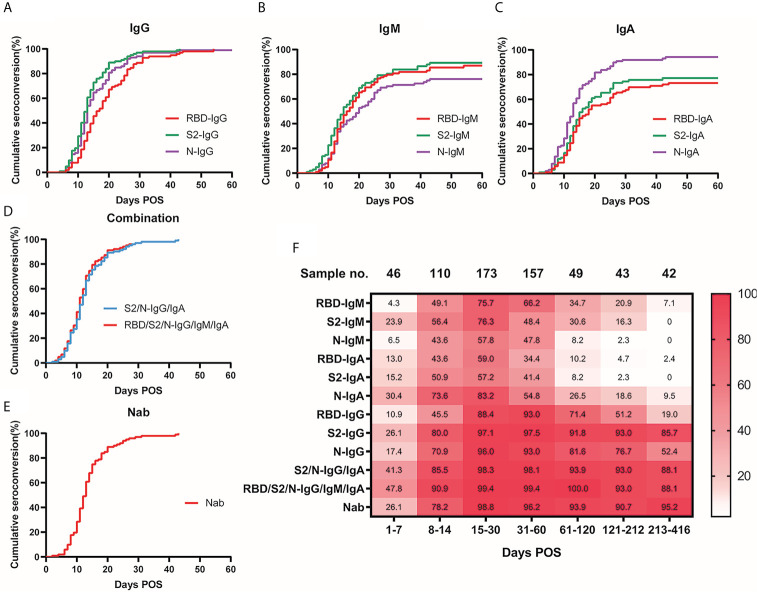
Seroconversion rate of antibodies and Nab over time. Cumulative seroconversion curve of S1-RBD, S2-ECD, and N-specific IgG **(A)**, (p = 0.0001), IgM **(B)**, (p = 0.0213), IgA **(C)**, (p < 0.0001). Days POS formed the x-axis and cumulative seroconversion rate formed the y-axis. The median seroconversion time (days) for antibodies targeting S1-RBD, S2-ECD, and N, respectively: IgG: 17, 13, 13; IgM: 16, 15, 20; and IgA: 18, 16, 13. Final seroconversion rate (%) for antibodies targeting S1-RBD, S2-ECD, and N, respectively: IgG: 96.1, 99.0, 98.0; IgM: 83.3, 85.3, 73.5; and IgA: 70.6, 73.5, 93.1. **(D)** Cumulative seroconversion curve of combination of S2/N-IgG/IgA (blue) and RBD/S2/N-IgG/IgM/IgA (red), the median seroconversion time was 12 and 11 d respectively. **(E)** Cumulative seroconversion curve of neutralizing activity (Nab titers ≥ detection limits of 1:20). Median seroconversion time of Nab was 13 d, and the final seroconversion rate was 99.0%. **(F)** Heatmap for visualization of the real-time positive rate of various specific immunoglobulins and neutralizing activity over time POS. Different shades of color indicate different positive rates (the darker the color, the higher the positive rate). Numbers in the color-block are the real-time positive rates of the corresponding time and antibodies. ECD, extracellular domain; Ig, immunoglobulin; N, nucleocapsid protein; Nab, neutralizing antibodies; POS, post onset of symptoms; RBD, receptor-binding domain; S, spike.

The cumulative seroconversion rates of specific antibodies are shown in **Figures 2A–C**. The cumulative curves of the same immunoglobulin against different antigens were significantly different (IgG: p = 0.0001; IgM: p = 0.0213, IgA: p < 0.0001). The three antigen-specific IgGs reached an almost 100% seroconversion rate around 30–45 d POS (S1-RBD: 96.1%, S2-ECD: 99.0%, and N: 98.0%), whereas IgM and IgA had lower cumulative positive seroconversion rates (IgM: 83.3%, 85.3%, and 73.5% for S1-RBD, S2-ECD, and N, respectively; and IgA: 70.6%, 73.5%, and 93.1% for S1-RBD, S2-ECD, and N, respectively). S2-IgG, N-IgG, and N-IgA had the same median seroconversion time of 13 d, which was the shortest among all SARS-CoV-2 immunoglobulins, followed by S2-IgM (15 d), S2-IgA and RBD-IgM (16 d), RBD-IgG (17 d), and RBD-IgA (18 d).

For the dynamic change of the seropositive rate (**Figure 2F**), the three antigen-specific IgGs were generally high, especially S2-IgG. N-IgA and S2-IgG had seropositive rates of 30.4% and 26.1%, respectively, which were the highest in the first week POS. At 30-61 d POS, IgA and IgM declined markedly. N-IgM, RBD-IgA, and S2-IgA positivity was less than 5% during 182–212 d POS (2.3%, 4.7%, and 2.3% respectively). During the 1-year follow-up time (213-416 d POS), most antibody positive rates dropped below 10%. However, S1-RBD, S2-ECD, and N specific IgG seropositive rates remained relatively high with a long duration. Notably, S2-IgG maintained a seropositive rate of 90.9% from 182 to 212 d POS and 85.7% from 213-416 d POS. These findings were consistent with previous dynamic curves in **Figures 1A–C**.

### Combining S2/N-Specific IgG/IgA Improved Detection Rate in Early COVID-19

The cumulative seroconversion rate of S2/N-IgG/IgA was not significantly different compared with to those of RBD/S2/N-IgG/IgM/IgA (**Figure 2D**), and the median seroconversion time of S2/N-IgG/IgA was 12 d POS, which was shorter than any of the single antibodies. Combining the four antibodies provided seropositive rates of 41.3% and 85.5% in the first and the second week POS, respectively, which were also much higher than those of the single antibodies (**Figure 2F**) .

### Nab Titer and Its Relationship With Immunoglobulins

Among the 11 viral isolates, the Nab titers were of highly consistent in the three serum samples (**Figures S3A, B**). The Nab titers of patient 02 with viral strain isolated from himself (ZJU-05) were not significantly higher or lower.

The dynamic change in the Nab titer started with a sharp increase in the early days POS, reaching its peak at 15–30 d, which is consistent with the other detected antibodies (**Figure 1D**). In addition, the distribution of different titers in each period varied (**Figure S4**). In the first week POS, in about a quarter of the samples, we detected a neutralizing titer in their serum. At 15–30 d POS, only 1.2% of the samples had lower than 1:20 Nabs (lower limit of detection). In the second month POS, the Nab positive rate was about the same as that during 15–30 d; however, the overall titer declined slightly. In the next 3-4 months POS, the overall titer declined further, and the highest proportions were 1:80 (30.6%) and 1:160 (28.6%). In the samples with the longest follow-up time (213-416 d POS), 95.2% samples still showed measurable Nabs, and the Nab positive rate was stable and no significant decrease was observed over the long follow-up time. The cumulative seroconversion rate of the Nabs is showed in **Figure 2E**. The median seroconversion time was 13 d POS, and the final seroconversion rate was 99.0%. The real-time positive rate for Nab activity is shown in **Figure 2F**, and the positive rate was similar to that of S2-IgG.

**Figure 3:**
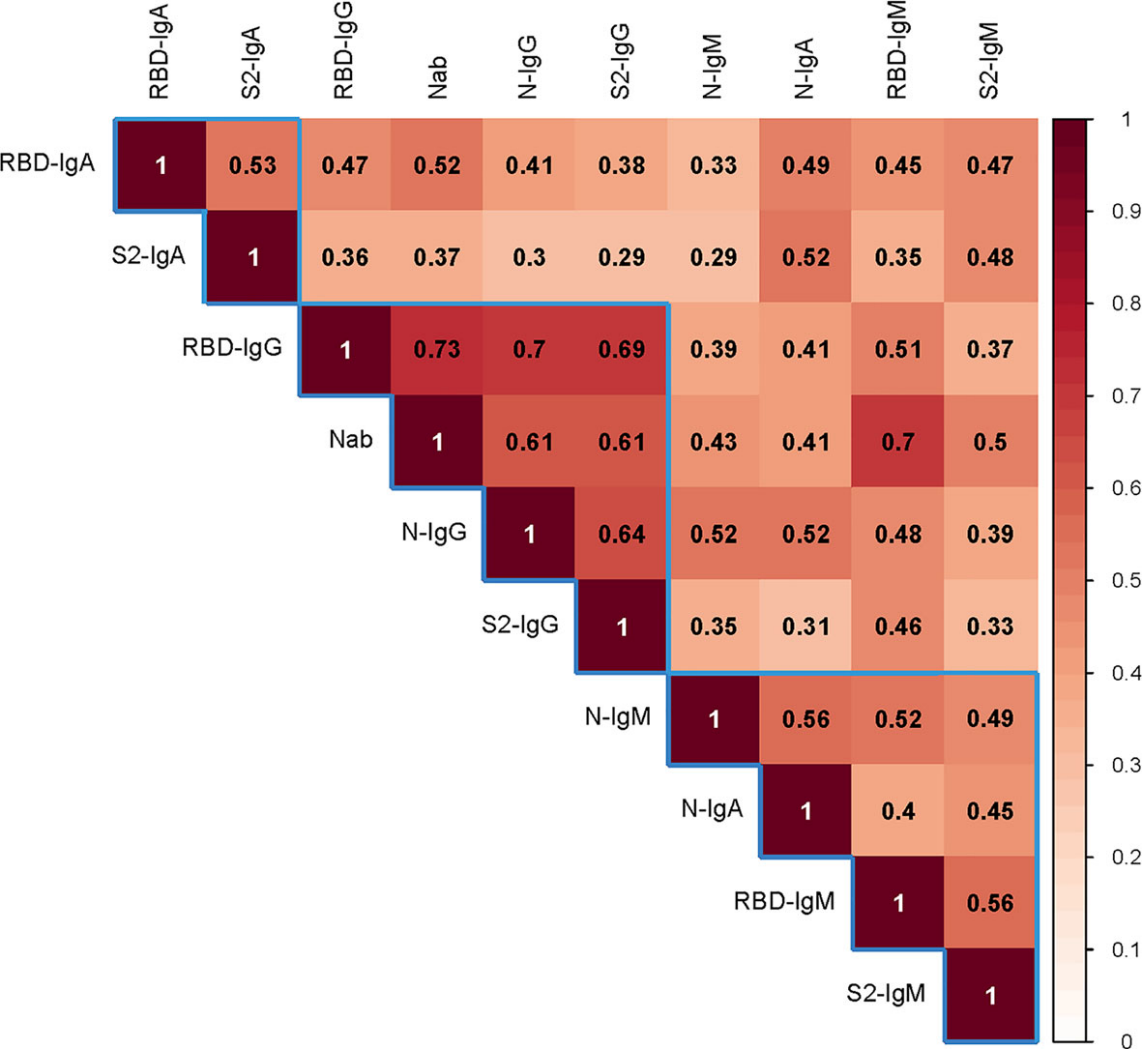
The correlation between the Nab titers and various antibodies. Spearman’s correlation test was used to examine the correlation between the Nab titer with various antibodies. Darker color indicates a stronger correlation. The number in the square is the relation value. The blue frame shows the clustering relationship. Ig, immunoglobulin; N, nucleocapsid protein; Nab, neutralizing antibodies; RBD, receptor-binding domain; S, spike.

The Nab titers were confirmed to correlate with all immunoglobulins including IgG, IgM, and IgA (**Figure 3**). S1-RBD-specific IgG correlated particularly with Nabs (R = 0.73, P < 0.001). Correlations between immunoglobulins were also significant, especially for IgG (N-IgG with S1RBD-IgG: R = 0.70, P < 0.001; N-IgG with S2-IgG: R = 0.63, P < 0.001; and S1RBD-IgG with S2-IgG: R = 0.69, P < 0.001).

### Nab Prediction From Tested Immunoglobulins With a Random Forest Plot

**Figure 4:**
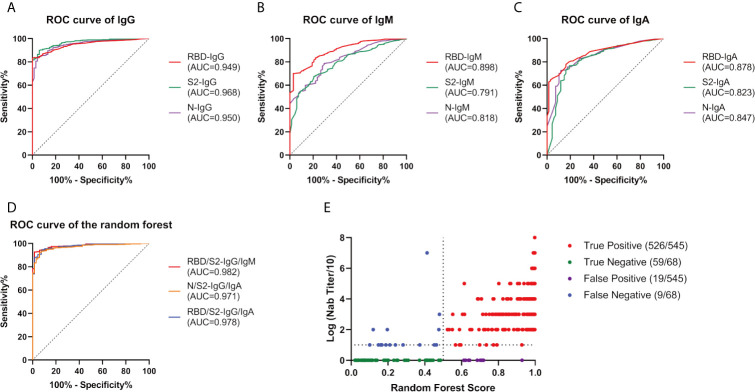
Random Forest efficacy with various indicators. We constructed a random forest model to predict the Nab titers (divided by 1:20) from the tested immunoglobulins. ROC curve of IgG **(A)**, IgM **(B)**, and IgA **(C)** as a single indicator. **(D)** ROC curve of the indicator combinations. **(E)** Performance of RBD/S2-IgG/IgM in the random forest plot, every dot represents a sample, red, green, purple, and blue represent true positive, true negative, false positive, false negative respectively. AUC, area under curve; Ig, immunoglobulin; N, nucleocapsid protein; Nab, neutralizing antibodies; RBD, receptor-binding domain; ROC, receiver operating characteristic; S, spike.

After data algorithm analysis, a random forest model was established based on QD-labeled LFIA to predict the Nab titers (divided by 1:20). Among all the single indicators, S2-IgG had a highest precision rate of 92.33%. Combining RBD/S2-IgG/IgM could best predict the Nab titers, with a precision rate of 95.43% (**Figure 4**).

### Different Clinical Conditions Reflected a Different Antibody Detection Status

**Figure 5:**
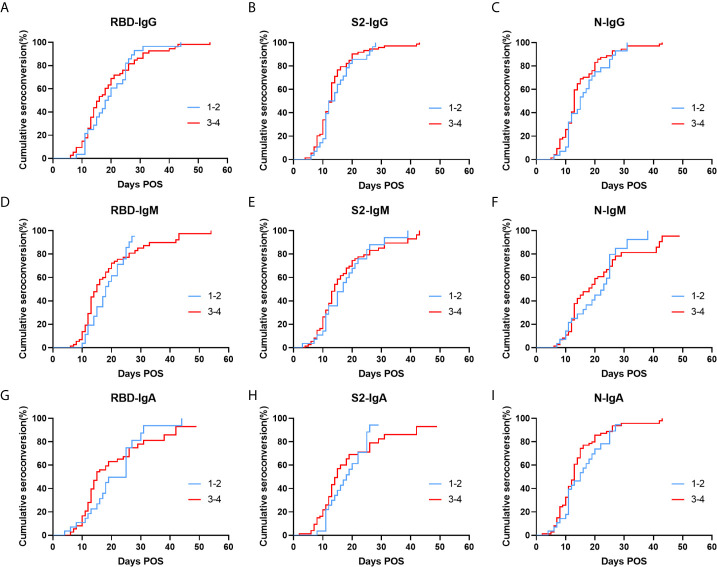
Cumulative seroconversion rates of RBD, S2, and N specific IgG **(A–C)**, IgM **(D–F)**, and IgA **(G–I)** in patients with different clinical types. Patients were divided into two groups, including low severity patients (blue) with mild and moderate severity and high severity patients (red) with severe and critically ill severity. P values were determined using the Log-Rank test. All P values in this figure are greater than 0.05. Ig, immunoglobulin; N, nucleocapsid protein; RBD, receptor-binding domain; S, spike.

The patients were divided into two groups according to the disease severity based on the China Diagnosis and Treatment Protocol for SARS-CoV-2 (version 7th), including low severity patients with mild and moderate severity and high severity patients with severe and critically ill severity. The cumulative seroconversion was achieved earlier in the high severity group, but there was no statistical difference (**Figure 5**). The median seroconversion times of Nab detection were 14 d and 12 d in less severe patients and more severe patients, respectively; and, the difference was also not significant (**Figure S5**).

In addition, patients with different viral shedding times had different antibody levels (**Figure S6**). Longer viral shedding time tend to result in higher antibody levels for N-IgG (p = 0.028) and N-IgM (p = 0.028).

For the effect of age on antibody production, there were significantly higher levels of RBD-IgG (p = 0.032), N-IgG (p = 0.023), N-IgA (p = 0.011) in patients who were 60 or older compared with that in younger patients (**Figure S7**). However, no significant difference was observed for the Nab titers in these two groups. We also found that the proportion of older men was greater among the more severe patients (**Figure S2**).

## Discussion

The present study revealed the kinetics of specific antibodies and neutralizing activities during the 1-year follow-up of COVID-19 patients. We confirmed the effective antiviral activity against live SARS-CoV-2 from 1-year inpatient followers, which is the result that gets the most attention. N-IgA was the most sensitive in the early stage of infection, while S2-IgG was present at a high level in the long time of observation. Combining S2 and N specific IgG and IgA increased the likelihood of being tested positive. Nabs correlated with all tested antibodies, particularly with S1-RBD specific IgG. The random Forest plot trained by the antibody data showed good accuracy in predicting Nab titers with RBD/S2-IgG/IgM.

The duration and effectiveness of antibodies are important for the SARS-CoV-2 pandemic control and vaccine development. A study from Wuhan, China has confirmed 9-month stability of neutralizing antibodies to SARS-CoV-2 in 39.8% people who were positive for pan-immunoglobulins, and Nab titers were lower in asymptomatic individuals (19). Our study stretched out the timeline to 1 year and also validated the existence and activity of anti-SARS-CoV-2 antibodies. However, the seroprevalence and titers of Nab is much higher in our study, this could be due to the only symptomatic individuals included.

We showed that specific immunoglobulins (IgG, IgM, IgA) targeting all detected antigens (S1-RBD, S2-ECD, N) were generally detected in patients with SARS-CoV-2, but reacted diversely. Both immunoglobulins and Nabs increase to a peak and then decreased during the follow-up period, which is similar to other acute viral infections (22). The varying responses of different antibodies suggested the best antigen for detection of specific immunoglobulins was S2 for IgG and IgM, and N for IgA. In that, S2-IgG and N-IgA were good indicators for SARS-CoV-2 antibody detection in the early phase and in the long term, respectively. This result indicated that N-IgA and S2-IgG have outstanding value in early diagnosis, and S2-IgG can be used as a long-term epidemiological marker. To assist clinical screening in practice, we used the combination of S2/N-IgG/IgA to elevate the likelihood of being tested positive, which requires only two strips of the LFIA assay (one for N-IgG/IgA, the other for S2- IgG/IgA).

Instead of IgM, IgA domination in the early course of infection was also found in a research from Sterlin et al. (23), who showed that IgA producing plasmablasts were the majority and contributed more during early neutralization than IgG and IgM. IgA ELISA was also shown to be highly sensitive (24). Usually, serum IgA is less noticed in antibody detection as it is a mucosal response antibody in respiratory infections. The IgA domination in the early course might be attributed to the earlier maturation of IgA as a mucosal antibody compared with IgG. Rapidly increases in N-specific IgA might also be caused by the large amount of N protein released by virus replication in the early stage of disease, resulting in massive exposure to antigen presenting cells. The rapidly decrease in N-IgA might reflect shuttling of IgA from the serum to the mucosa to exert its mucosal immunity effect and its short half-life. This finding may challenge the traditional screening by IgM and IgG.

The robust reaction of S2 was unexpected, because S2 is not assessed routinely in most serological tests. Original assays usually select full-length spike, S1, S1-RBD, and N as detection antigens (25). This finding shifted the focus to S2, a SARS-CoV-2 structural protein that mediates membrane fusion of the virus and host cells, thus facilitating viral invasion. S1 is highly glycosylated and forms an S1/S2 complex, which promotes prefusion. After the dissociation of S1, S2 is exposed and forms a post-fusion spike on the virion (12). In addition, we found in our previous study that the post-fusion state naturally exists without the fusion process (26), resulting in more S2 than S1. This might make S2 more accessible and generate more S2-specific antibodies. In addition, we suspect that because S1 has more glycosylation sites than S1, it promotes immune escape by shielding specific epitopes from antibody neutralization, so S2 produces more antibodies (27) Furthermore, a study found preexisting predominant IgG to SARS-CoV-2 that targeted S2, and the cross-reactive antibodies could effectively neutralize both authentic and S pseudotypes of SARS-CoV-2 (28). These findings prompted us to develop a universal vaccine targeting CoVs, taking advantage of the conserved S2. A study has already used S2 or its functional segments as targets to prevent infection and obtained good results (29). Although S2 antigen has not been used as a vaccine, we believe that S2 could be used as a vaccine complement or detection target. We could also use the S2-specific antibody in the recently proposed antibody cocktail therapy to limit the escape of viral mutants (21, 30).

Surprisingly that IgM positivity appeared later than IgG positivity, and the level was relatively low, which was not consistent with our understanding of the antibody response. However, it was consistent with the study from Sterlin et al. (23), which reported only small population of IgM producing plasmablasts in the early stages of SARS-CoV-2 infection. It might also be attributed to the relatively low sensitivity of IgM detection.

The correlation between IgM and Nab titers was not as good as that between IgG and Nab titers, which was consistent with previous research (16, 31). However, S2 was not considered in their research. We noted that IgA had a lower correlation with Nab than IgM. Moreover, S1-RBD specific antibodies had a higher correlation with Nab than S2 or N, which supported RBD’s immunodominance in neutralization (32). However, unlike Sterlin et al. (23), we did not purify the immunoglobulins; therefore, the Nab titers we tested were an effect of pan-immunoglobulins as well as serum nonspecific components such as interferon, making our reported correlations not so direct.

Rapid diagnosis is advantageous for screening and follow-up treatment. We used the QD-labeled LFIA method to detect the antibody levels. This method produces results rapidly (~10 minutes), and hundreds of samples can be detected within a portable fluorescence detector in 1 hour. The use of serum samples reduces the exposure risk of healthcare worker compared with sampling of sputum and throat swabs. More importantly, this technique has high sensitivity and specificity. The trained random Forest plot performed well to predict whether the Nab titer is high or low, which saves time compared with performing a neutralizing test using authentic virus, which should proceed in a biosafety level III laboratory. Thus, QD-labeled LFIA is a good POCT device to detect specific antibodies quantitatively, allowing screening of infector humoral immunity and to detect post-vaccine immunity.

Our study had several limitations. First, because of the short linear range of the detection results of QD-labeled LFIA, we were not able to transfer all the T/C values into actual antibody concentration. Second, during the early emergency situation, we could not obtain more frequent follow-up serum samples to meet the scientific research needs, thus delaying the seroconversion date. Third, at the early stage of the pandemic, we did not know that SARS-COV-2 could be spread through feces; therefore, there was no testing of fecal nucleic acids and no analysis of fecal nucleic acid with antibodies. Fourth, although we surveyed the correlation between different antibodies and Nab titers, we did not determine the exact protection effect of the different antibodies because we tested the total Nab titers, not single antibody effects.

An understanding of the temporal dynamics of protective immunity is vital, and will be important to determine the overall course of the pandemic and post-pandemic dynamics. The novel coronavirus SARS-CoV-2 firstly reported in Wuhan, China generated a cohort of patients who had the longest days POS worldwide. In this study, we detected specific antibodies and Nab activity in sequential SARS-CoV-2 patients during hospitalization and follow-up to exceed 1 year POS. The results illustrate the diverse dynamics of different antigen-specific antibodies and revealed the neutralizing activity in the acute and convalescent phases of infection. The characteristics of S2 and IgA might be considered in screening and vaccine development.

## Data Availability Statement

The raw data supporting the conclusions of this article will be made available by the authors, without undue reservation.

## Ethics Statement

The studies involving human participants were reviewed and approved by The Ethics Committee of The First Affiliated Hospital, Zhejiang University School of Medicine (ethical approval no. 2020IIT-255). Written informed consent from the participants’ legal guardian/next of kin was not required to participate in this study in accordance with the national legislation and the institutional requirements.

## Author Contributions

DS, TW, JW, and CD were co-first authors of this manuscript. HY, MZheng, NW, and ZC conceived and designed this study. HY, DS, TW, JW, CD, XL, LC, FL, ZW, and MZhou performed the experiments. QC, DS, and ZC prepared the strip of QD-labeled LFIA. ZC, HY, MZheng, RL, KC, HW, CJ, and MG collected and analyzed the data. HY, MZheng, JW, and DS wrote the manuscript. All authors contributed to the article and approved the submitted version.

## Funding

This work was supported by Zhejiang Provincial Key Research and Development Program (#2021C03043), National Science and Technology Major Project for the Control and Prevention of Major Infectious Diseases in China (#2018ZX10102001, #2018ZX10711001), and Zhejiang Provincial Scientific Research Project (LGF21H180008).

## Conflict of Interest

One author has declared that the following interests are relevant to the submitted work. QC is an employee of Hangzhou Chemi Health Technology Co., Ltd. He prepared the strip of QD-labeled LFIA and had nothing to do with the detection and data analysis.

The remaining authors declare that the research was conducted in the absence of any commercial or financial relationships that could be construed as a potential conflict of interest.
