# Supplementary material for: Dynamic Characteristic Analysis of Antibodies in Patients With COVID-19: A 13-Month Study
Source: Front Immunol. 2021 Jul 20;12:708184. doi: 10.3389/fimmu.2021.708184 (PMC8330131; doi:10.3389/fimmu.2021.708184)
Supplement: Supplementary file 1 [file DataSheet_1.docx]

***Supplementary Material***

**1 Supplementary Figures and Tables**

**1.1 Supplementary Figures**


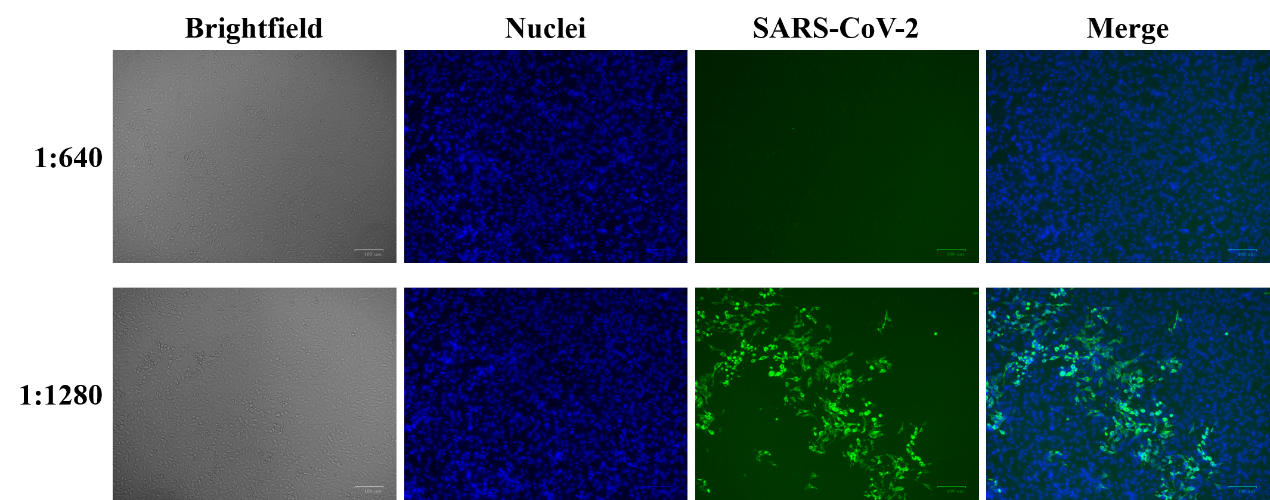


**Supplementary Figure 1.** Immunofluorescence microscopy images from a serum sample with a Nab titer of 1:640.





**Supplementary Figure 2**. **Demographic distributions of patients with different clinical types.** Patients with different clinical severity (mild, moderate, severe, and critically ill) were separated with age and sex. The number of patients form the y-axis.


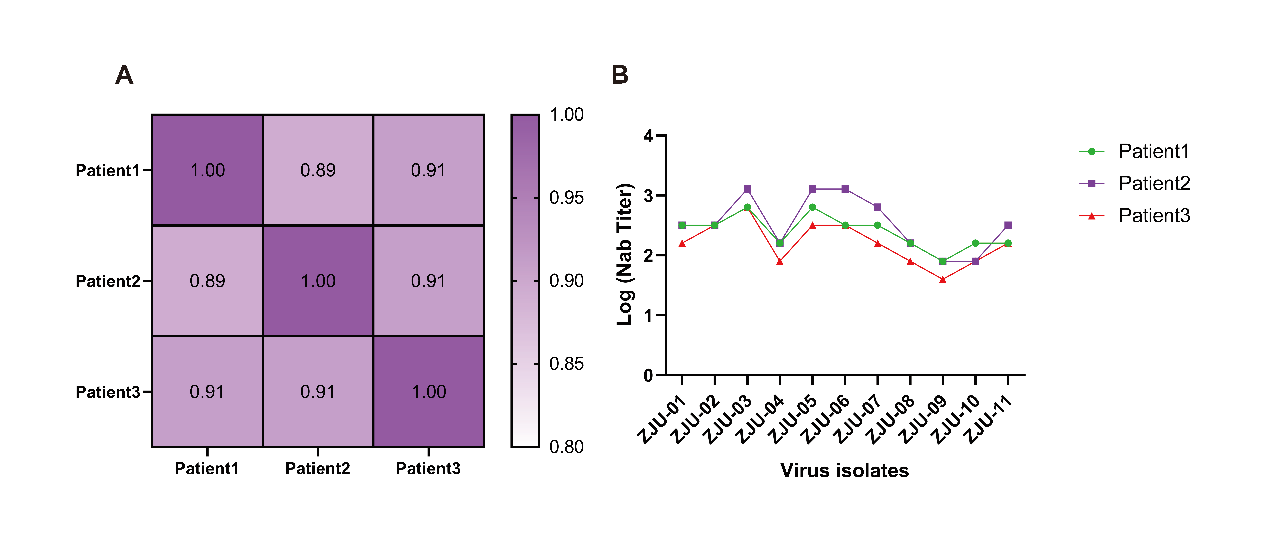


**Supplementary Figure 3**. **Nab titers of 11 viral isolates in samples from three patients**. **(A)**, Correlation of sera Nab titers from 3 patients among different virus isolates (all P values < 0.05). **(B)**, The Nab titers of sera from 3 patients among different virus isolates.


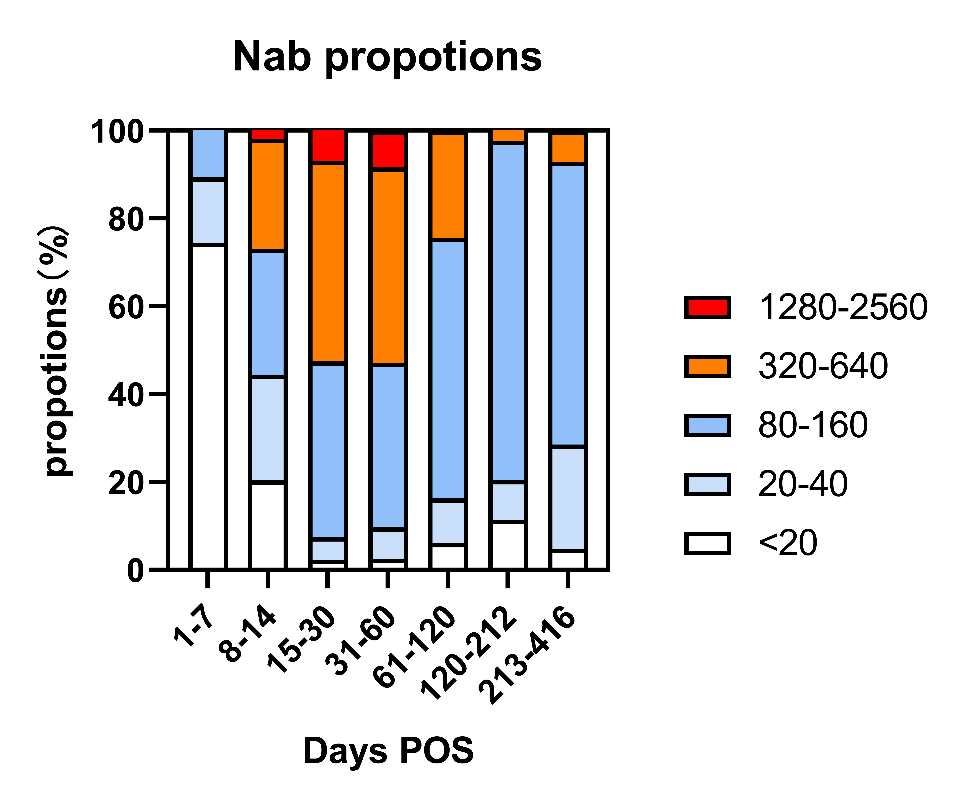


**Supplementary Figure 4**. **Proportions of Nab titers at different time periods.** Distribution of Nab titers from different period of days since symptom onset were showed with different colors.


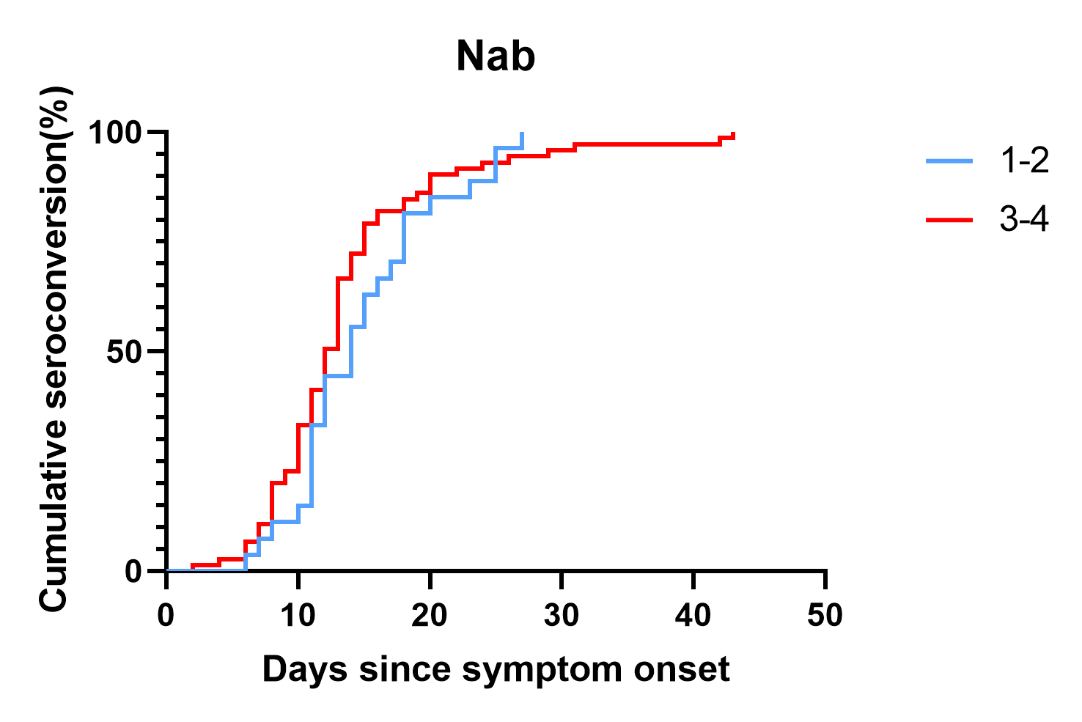


**Supplementary Figure 5**. **Cumulative seroconversion rate (%) of Nab activity in patients with different clinical types.** Patients were divided into two groups, including low severity patients (blue) with mild and moderate severity and high severity patients (red) with severe and critically ill severity. The median seroconversion times of Nab detection were 14 d and 12 d in less severe patients and more severe patients, respectively (P > 0.05).


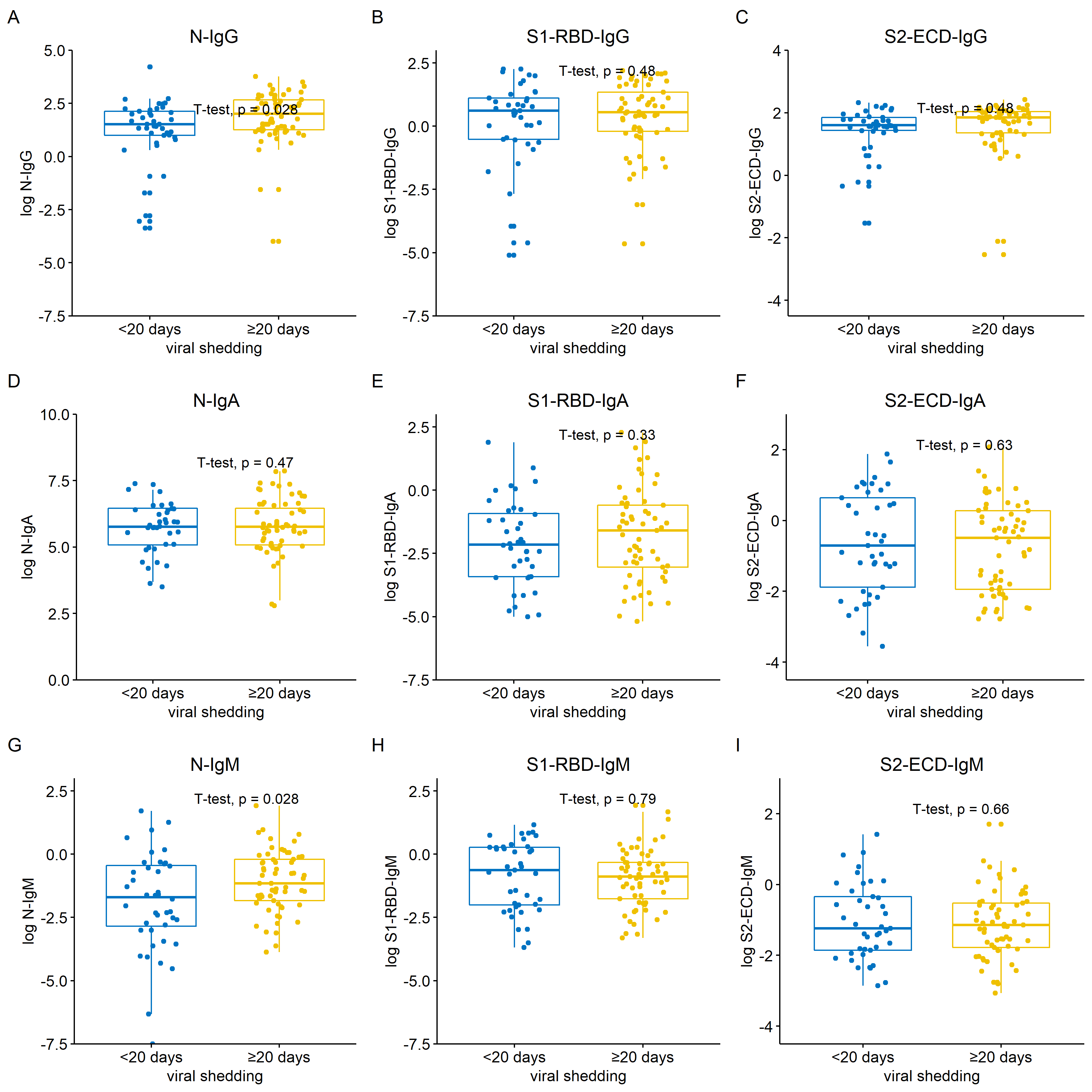


**Supplementary Figure 6. Variations in antibody levels at different viral shedding times.** Patients are divided into two groups according to their median viral shedding time (20 d). The maximum values of N, RBD, S2 specific IgG (**A-C**), IgA (**D-F**), and IgM (**G-I**) were compared in these two groups. P values were determined using Student’s t- tests.


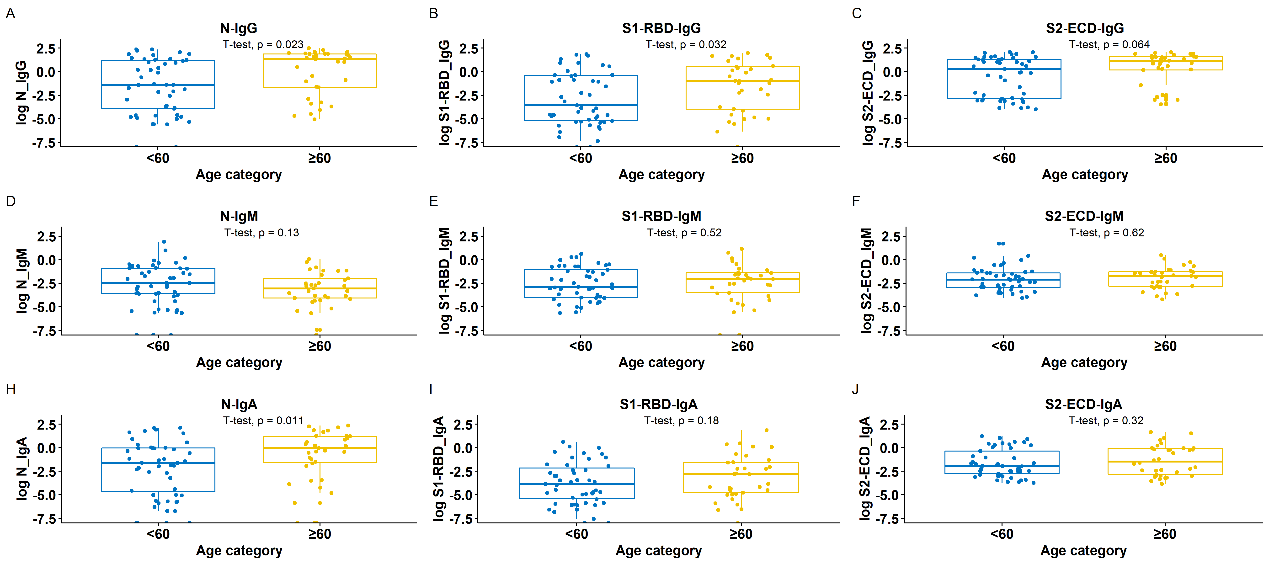


**Supplementary Figure 7. Variations in antibody levels for different ages.** Patients are divided into two groups according to their age (60 y). The maximum values of N, RBD, S2 specific IgG (**A-C**), IgA (**D-F**), and IgM (**G-I**) were compared in these two groups. P values were determined using Student’s t- tests.

**1.2 Supplementary Tables**

**Supplementary Table 1:** Patient and sample number in each different time period

| Time period (days POS) | 1-7 | 8-14 | 15-30 | 31-60 | 61-120 | 121-212 | 213-416 |
| --- | --- | --- | --- | --- | --- | --- | --- |
| Patient number | 35 | 73 | 88 | 80 | 46 | 43 | 42 |
| Sample number | 46 | 110 | 173 | 157 | 49 | 43 | 42 |

**Supplementary Table 2:** Cut off value and specificity of LFIA

|  | **S1-RBD** | | | **S2-ECD** | | | **N** | | |
| --- | --- | --- | --- | --- | --- | --- | --- | --- | --- |
| Antibodies | Serum cut off | Plasma cut off | Specificity  (%) | Serum cut off | Plasma cut off | Specificity  (%) | Serum cut off | Plasma cut off | Specificity  (%) |
| IgG | 0·1 | 0·1 | 100 | 0·116 | 0·199 | 100 | 0·1 | 0·1 | 100 |
| IgM | 0·1 | 0·184 | 97 | 0·101 | 0·185 | 100 | 0·1 | 0·12 | 98 |
| IgA | 0·05 | 0·05 | 97 | 0·214 | 0·092 | 100 | 0·05 | 0·05 | 100 |
